# Chemotropic vs Hydrotropic Stimuli for Root Growth Orientation in Microgravity

**DOI:** 10.3389/fpls.2019.01547

**Published:** 2019-11-22

**Authors:** Luigi Gennaro Izzo, Leone Ermes Romano, Stefania De Pascale, Giacomo Mele, Laura Gargiulo, Giovanna Aronne

**Affiliations:** ^1^Department of Agricultural Sciences, University of Naples Federico II, Portici, Italy; ^2^Institute for Agricultural and Forest Systems in the Mediterranean, National Research Council, Ercolano, Italy

**Keywords:** chemotropism, *Daucus carota*, hydrotropism, microgravity, root tropisms, X-ray microtomography

## Abstract

Understanding how plants respond to spaceflight and extraterrestrial environments is crucial to develop life-support systems intended for long-term human explorations. Gravity is a main factor influencing root development and orientation, typically masking other tropisms. Considering that reduced levels of gravity affect many plant responses in space, the interaction of other tropic stimuli in microgravity represents the frontier to be investigated aiming at life-support systems optimization. In this paper we report on MULTITROP (Multiple-Tropism: interaction of gravity, nutrient and water stimuli for root orientation in microgravity), an experiment performed on the International Space Station during the Expedition 52/53. Scientific aim of the experiment was to disentangle hydrotropism from chemotropism for root orientation in absence of the gravity stimulus. Among several species relevant to space farming, *Daucus carota* was selected for the experiment because of its suitability with the experimental hardware and setup. At launch site, carrot seeds were placed between two disks of inert substrate (one imbibed with water and the other with a disodium phosphate solution) and integrated into a hardware developed, refurbished and flight-certificated by Kayser Italia. Post-flight, a Ground Reference Experiment was performed. Root development and orientation of seedlings grown in microgravity and at 1g condition were measured through 3D-image analysis procedures after imaging with X-ray microtomography. Radicle protruded preferentially from the ventral side of the seed due to the asymmetric position of the embryo. Such a phenomenon did not prevent the achievement of MULTITROP scientific goal but should be considered for further experiments on radicle growth orientation in microgravity. The experiment conducted in space verified that the primary root of carrot shows a positive chemotropism towards disodium phosphate solution in the absence of the gravity stimulus. On Earth, the positive chemotropism was masked by the dominant effect of gravity and roots developed downward regardless of the presence/absence of nutrients in the substrate. Taking advantage of altered gravity conditions and using other chemical compounds, further studies should be performed to deepen our understanding of root chemotropic response and its interaction with other tropisms.

## Introduction

Plants are sessile organisms, but not necessarily motionless. Indeed, different plant organs typically show directional growth responses, referred to as tropisms, which are driven by directional stimuli (Gilroy, 2008). Plant movements have fascinated scientists all over the world since the early studies of Charles Darwin in the late 1800s, when he and his son Francis minutely studied plant tropisms in response to external directional stimuli (Darwin and Darwin, 1881). Later, numerous studies have highlighted the presence of various plant tropisms including those widely recognized and new discoveries such us phonotropism (Esmon et al., 2004; Gagliano et al., 2017). Most tropic responses follow the almost centenary Cholodny–Went theory (Went, 1926; Cholodny, 1940), and, more recently, the molecular mechanisms behind these processes have been defined (Kleine-Vehn et al., 2010; Harmer and Brooks, 2018). However, deepening the study of tropisms new challenges have arisen as those trigged by the evidence that hydrotropism does not follow the Cholodny–Went theory (Shkolnik et al., 2016).

Although gravitropism is dominant on Earth, roots have also evolved other specific tropisms to explore the soil in search of beneficial environmental conditions (Harmer and Brooks, 2018). Root can direct growth toward environments with higher water availability through a positive hydrotropism (Dietrich, 2018). Differently, chemotropism is more questionable and chemotropic responses can be ambiguated. A negative chemotropism in avoiding detrimental concentrations of (NaCl) salts is defined as halotropism (Sun et al., 2008). Although a species with positive halotropism has been identified (*Bassia indica* Wight) (Shelef et al., 2010), halotropism can be considered as a general response to avoid stress conditions and not as a search for specific nutrients. To our knowledge, clear evidence for directional growth of roots toward a chemical compound has been found only a century ago (Newcombe and Rhodes, 1904). A positive bending response toward disodium phosphate (Na_2_HPO_4_) was observed in roots of *Lupinus albus* L. when medium containing 0.28% Na_2_HPO_4_ was attached to one side of the root tip. Higher concentrations of 1% or 1.5% resulted in the same directional growth response, followed by the roots dying off. In addition, roots of *L. albus* did not display directional growth toward other salts typically absorbed by plants as food.

While progresses have been made in understanding plant tropisms, many more questions remain open. In addition, much knowledge regarding plant tropisms is related to *Arabidopsis thaliana* L. and still remains questionable if other species behave in the same way. Still, a better knowledge on root growth strategies and tropism interactions is required to improve plant cultivation in space and implement bioregenerative life support systems (Lasseur et al., 2010). Research on plant tropisms has become increasingly popular especially in the framework of developing systems for cultivation in space. In microgravity, the possibility to cut down the effect of gravity in root growth orientation has encouraged research on root tropisms also providing novel results (Millar et al., 2010; Vandenbrink et al., 2016).

Nowadays, the use of non-destructive and non-invasive techniques such as the X-ray microcomputed tomography (X-ray microCT) has become increasingly widespread in the study of root development. Gregory et al. (2003) applied this technique to monitor wheat seedling establishment in soil. More recently, X-ray microCT has been used to test the effect of different seed enhancement technologies on the germination of sugar beet seeds in soil (Blunk et al., 2017), to monitor the effects of soil medium on root system development of corn seedlings (Subramanian et al., 2015), and to measure root traits in barley seedlings (Hargreaves et al., 2009).

In this paper we report on MULTITROP (Multiple-Tropism: interaction of gravity, nutrient and water stimuli for root orientation in microgravity), an experiment performed on the International Space Station (ISS) during the Expedition 52/53, funded and coordinated by the Italian Space Agency (ASI) which also provided access to the space resources thanks to a bilateral agreement with NASA.

The scientific aim of the MULTITROP experiment was to disentangle the role of gravity from two other stimuli for root orientation: hydrotropism and chemotropism. Among several species relevant to space farming (Mitchell, 1994), *Daucus carota* L. was selected for the experiment because of its suitability with the experimental hardware and with the estimated timeline from sample integration at launch site to payload arrival on the ISS. We hypothesized that, in the absence of the gravity stimulus, a positive chemotropism of roots would prevail over hydrotropism, by inducing a directional growth toward the nutrient solution. Moreover, we applied X-ray microCT and 3D image analysis in order to compare the morphology of the radicles grown in microgravity and on Earth.

## Materials and Methods

### Plant Materials

Seeds of *D. carota* cv Chantenay by Franchi Sementi were used for this study. Species selection was performed applying the method of subsequent excluding criteria to a set of 50 crop species (Aronne et al., 2018). Carrot seeds showed the best adaptability to the hardware characteristics and the experimental timeline scheduled for the spaceflight experiment. Germination percentage of the seed batch was tested in dark conditions at a temperature of 22°C sowing a total of 250 seeds distributed in five petri dishes lined with wet filter paper.

### Seed Morphology and Radicle Protrusion

**Figure 1:**
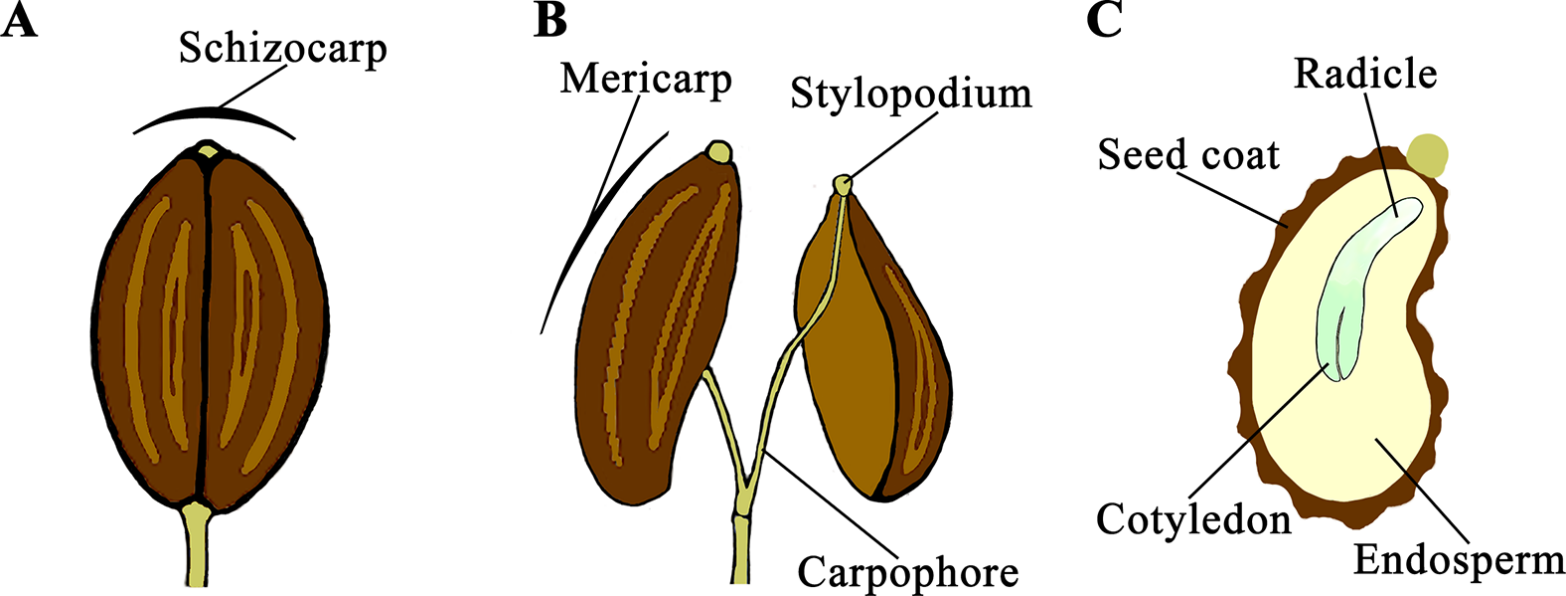
Seed development and morphology in *Daucus carota*. After pollination, a schizocarp develops from the ovary **(A)** and consists of two mericarps attached to a carpophore through the stylopodium **(B)**. Mericarps are curved and contain the endosperm and the embryo with the radicle pointing toward the ventral side **(C)**.

*D. carota* is a species belonging to the Apiaceae family that is characterized by umbel inflorescences of many hundreds of minute flowers. Single flowers have an inferior ovary, separated in two locules each containing one anatropous ovule. Externally there are two styles swollen at the base forming a stylopodium. After pollination, the ovary develops into a dry fruit (schizocarp) consisting of two mericarps attached to a central axis (carpophore) (**Figure 1A**). At maturity, the schizocarp splits and the two mericarps remain attached to the carpophore through the stylopodium (**Figure 1B**). Carrot mericarps are curved, covered with a spiny coat and commonly referred to as “carrot seeds.” Internally most of the volume contains the endosperm and the embryo with the radicle pointing toward the ventral side (**Figure 1C**).

**Figure 2:**
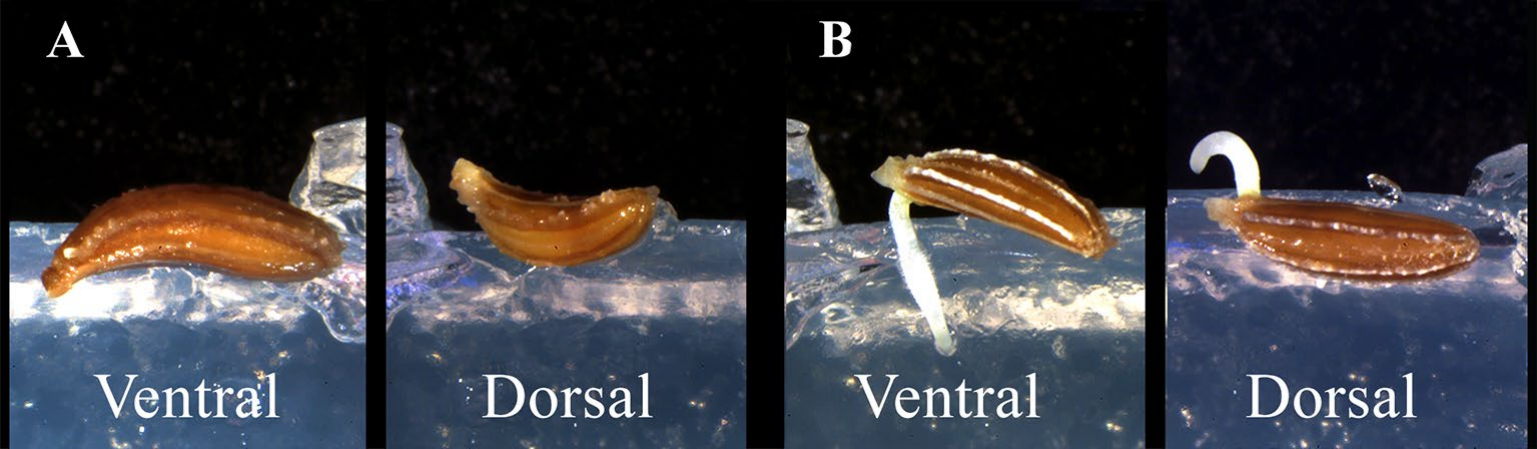
Two different seed orientations tested to evaluate radicle protrusion. **(A)** seeds placed in ventral position (V), with the radicle pointing downwards, and in dorsal position (D), with the radicle pointing upwards. **(B)** radicle protrusion of carrot’s seeds in V and D orientation.

The asymmetry of carrot seeds (both as overall shape and embryo position), led us to deepen the relation between seed position and modes of radicle protrusion at germination. Considering that such insights were not available on literature, before planning the space experiment, we carried out closeup observation tests aimed to describe the effect of embryo position on radicle protrusion during early stage of seed germination. Before sowing, carrot seeds were surface sterilized in 3% (v/v) sodium hypochlorite for 5 min and then rinsed with sterile water. Germination tests were carried out in dark conditions at a temperature of 22°C on 0.8% agar gel in petri dishes. Two different seed orientations were tested to evaluate radicle protrusion. The seeds were placed both in ventral position (V), with the radicle pointing downwards (according to the gravity vector), and in dorsal position (D), with the radicle pointing upwards (opposite to the gravity vector) (**Figure 2A**). Seed germination and radicle protrusion were monitored daily by means of a digital camera (EOS 60D, Canon, Tokyo, JP) mounted on a dissecting microscope (SZX16, Olympus Inc., Tokyo, JP).

### Spaceflight Experiment

**Figure 3:**
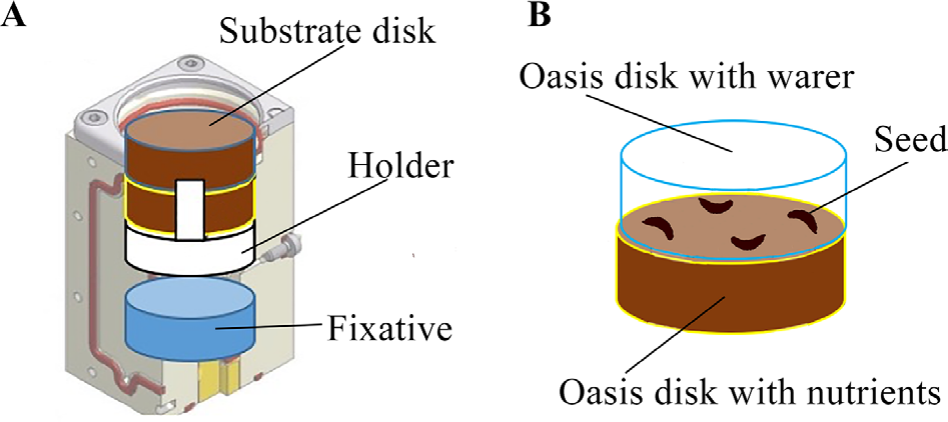
Experimental setup of the MULTITROP experiment within the hardware provided by Kayser Italia. Each culture chamber is equipped with a fixative reservoir and has been implemented with two substrate disks and a substrate holder **(A)**. The seeds were placed between two substrate disks imbibed with either pure water or nutrient solution **(B)**.

Spaceflight experiment was conducted on the ISS in a refurbished hardware: a BIOKON container equipped with two YING-B2 experimental units (EUs) previously flown for the YING experiment supported by the European Space Agency in 2009. Both the BIOKON and the YING-B2 units have been designed, manufactured and certified for launch by Kayser Italia. Each YING-B2 consisted of four culture chambers (CCs) each of which equipped with a fixative reservoir (**Figure 3A**). RNALater (Sigma-Aldrich, St. Louis, Missouri, US) was used as chemical fixative. The CCs have been implemented with a 3D-printed holder to accommodate two substrate disks (Oasis^®^ Growing Solutions, The Netherlands) imbibed with either pure water (W) or a nutrient solution (N) (**Figures 3A, B**). In each CC four seeds were placed in between the two substrate disks.

More specifically, following Newcombe and Rhodes (1904) a solution containing 0.28% Na_2_HPO_4_ was used to test chemotropic response of carrot roots. Once imbibed, substrate disks where centrifuged at 6*g* for 10 min to prevent possible leakage due to hypergravity conditions expected during launch operations. It was ascertained with specific tests that the amount of water/nutrient solution retained by the substrate upon centrifugation was adequate to activate germination (seed imbibition, radicle protrusion) and root growth. Before flight, carrot seeds were surface sterilized as reported above. Four seeds were then placed between the two substrate disks per each CC (**Figure 3B**), for a total of 32 seeds tested during the experiment in microgravity. Sowing was performed under a dissecting microscope to ensure that the four seeds in each CC were placed alternating dorsal and ventral orientation which refer to radicle expected to protrude toward W or N disk, respectively.

The MULTITROP payload was launched to the ISS on SpaceX CRS-13 (December 15th, 2017). After 175 h from seed imbibition, the US astronaut Scott David Tingle stopped the biological processes by activating the injection of the RNALater fixative and therefore concluded the experiment. The payload was stored at ISS node 2 temperature until returned *via* CRS-13 (January 13th, 2018), and was sent to the science team laboratory for post-flight analyses.

### Ground Reference Experiment

After the accomplishment of the experiment on the ISS, a Ground Reference Experiment (GRE) on Earth was performed using the same hardware and the same batch of the seeds used to perform the experiment on the ISS. Experiment was setup as previously reported for spaceflight experiment, using a BIOKON container and two YING-B2 EUs. To compare spaceflight experiment and GRE, we performed the control test in a growth chamber simulating the temperature regime recorded during the flight experiment and the same experiment duration. Data recorded on the ISS during the experiment showed that temperature ranged between 22 and 26°C, and was in average 24°C. The GRE was performed with two different setups obtained according to the gravity vector orientation of the Oasis disks: A) N disk at the bottom and W at the top, B) W disk at the bottom and N at the top.

### X-Ray Microtomography

Oasis foam is an opaque material, therefore, to analyze root orientation and seedling morphology without dismantling the CC setup, X-ray microCT of the scanned unit (substrate disks, still mounted in their holders, and the germinated seeds), was used for 3D imaging of the biological samples. All substrates from the spaceflight experiment and half those from the GRE experiment were scanned by a Skyscan 1272 desktop microtomograph (Bruker, US). For each scanned unit, a three-step washing with distilled water was first performed in order to dilute the fixative inside the substrates because it prevented from obtaining the image contrast required for displaying radicles. Scans of each unit were performed at 69 mm distance to the X-ray source that was set at 50 kV voltage and 200 µA current. For each sample 322 projection images, one each 0.6 deg rotation step, were acquired by six frames averaging at 17-micron resolution and 171 ms of exposure time. Volume of the samples was obtained using NRecon software v1.7.1 (Bruker, US), by means of 776 cross sectional images, each having size of 1,224 × 1,224 pixels. The beam hardening and the ring artifact correction procedures were applied for the final volume reconstruction. This latter consisting of 17-micron sized cubic voxels. 3D images were processed applying mathematical morphology operators and finally analyzed by means of 3D image analysis procedures using CTAn software v1.16.4 (Bruker, US). Besides the seed volumes, for each sprouted radicle, the following parameters were calculated: radicle volume, radicle mean diameter, radicle length and radicle tortuosity, which is the ratio between the radicle length and the Euclidean distance between the radicle ends. In detail, radicle volume was calculated as the sum of voxel volumes representing the radicle, the radicle mean diameter was obtained as the mean of the radicle thickness distribution determined by the “successive opening” algorithm (Dougherty et al., 2003), the radicle length was determined after “skeletonization” of the radicles’ images (Ballard and Brown, 1982).

Radicles were scored as N or W according to the location of the root tip in the substrate imbibed with disodium phosphate solution or pure water, respectively. The same was applied to hypocotyls, scored as H, if developed.

### Data Statistical Analyses

Seed germination on Earth and ISS was compared using a Chi-square test. For all tests performed, root growth direction was analyzed by using a Chi-square test hypothesizing that the expected frequencies for root tip location were 100% in the substrate imbibed with Na_2_HPO_4_ (N). In the case of p < 0.05, the observed frequencies differ from those expected, therefore not showing a preferential growth of roots toward N. The influence of gravity (i.e., 1g and microgravity) on root volume, root mean diameter, root length, and root tortuosity was analyzed by using independent samples *t*-test. Significance was accepted as *P* <0.05. All data were processed using Microsoft Excel and STATISTICA ver. 8.0 (StatSoft, Inc. 2008).

## Results

The hypothesis that carrot seeds could have a preferential side for radicle protrusion was proved. On Earth, radicle of seeds in dorsal orientation protruded upwards and later curved downwards to the gravity vector (**Figure 2B**). Conversely, radicle protruded immediately downwards in ventral orientation (**Figure 2B**). The phenomenon of radicle emergence from the ventral side of the seed, was taken into account to setup the spaceflight experiment and GRE. We considered that in absence of gravitropic stimulus root growth direction could be biased by seed orientation at sowing. According to these results, we decided to avoid any preferential orientation by placing half of the 32 seeds with the embryo position facilitating the radicle growth into the substrate with the nutrient solution (Rn) and the remaining half into water (Rw).

During the experiment performed in microgravity, on a total of 32 seeds, 27 germinated. Such a rate (84%) corresponded to the average germination rate (83.6%) of the seed batch that we have used for tests on Earth. Among the germinated seeds, 16 were those positioned as Rn and 11 as Rw. We analyzed data assuming a positive chemotropic response of roots in microgravity and, thus, we tested the hypothesis that germinated seeds developed the radicle into the N substrate, independently from the embryo position at sowing. Results of the Chi-square test showed that the difference between the observed and expected values are less than the critical value when data are pooled together, and also when they are separated into the two categories of seed orientation (**Table 1**). The great majority of the Rn seeds (15 out of 16) developed the root in the substrate with nutrient solution, showing that the observed results match those expected. The occurrence of chemotropism was also verified in seeds with embryo position facilitating root growth into the substrate with water (Rw). In such a case, six out of eleven seedlings oriented their radicle growth toward the nutrient solution. These data proved that radicles of carrot seedlings presented positive chemotropism in microgravity.

**Table 1:** Chi-square analysis of root growth direction of *Daucus carota* in 1g (GRE) and in microgravity (ISS) conditions.

	Substrate	Seed	Expected (%)		Observed	(%)	x^2^		
	Orientation	Orientation	N	W	N	W			
ISS		Rn	100	0	94	6	0.06	1	0.80
		Rw	100	0	55	45	2.27	1	0.13
		Rn + Rw	100	0	78	22	1.30	1	0.24
GRE	A	Rn + Rw	100	0	100	0	0.00	1	1.00
	B	Rn + Rw	100	0	0	100	29.00	1	0.00

Rn and Rw refer to seed orientation facilitating the radicle growth toward the substrate with nutrients (N) or with water (W), respectively. A = seeds placed with the radicle pointing downwards; B = seeds placed with the radicle pointing upwards.

In the GRE, 28 out of the total 32 seeds germinated (87.5%) and this value does not differ from that achieved in microgravity (*P* = 0.72). Under 1g conditions, gravitropism was dominant on both chemotropism and hydrotropism and roots always developed downward regardless of the presence/absence of nutrients in the substrate and of the embryo position. Results of the Chi-square test showed that all seeds (Rn and Rw) directed root growth into the substrate with nutrient solution when it was placed below the seeds and, similarly, all into the substrate with water when it was placed below the seeds (**Table 1**).

**Figure 4:**
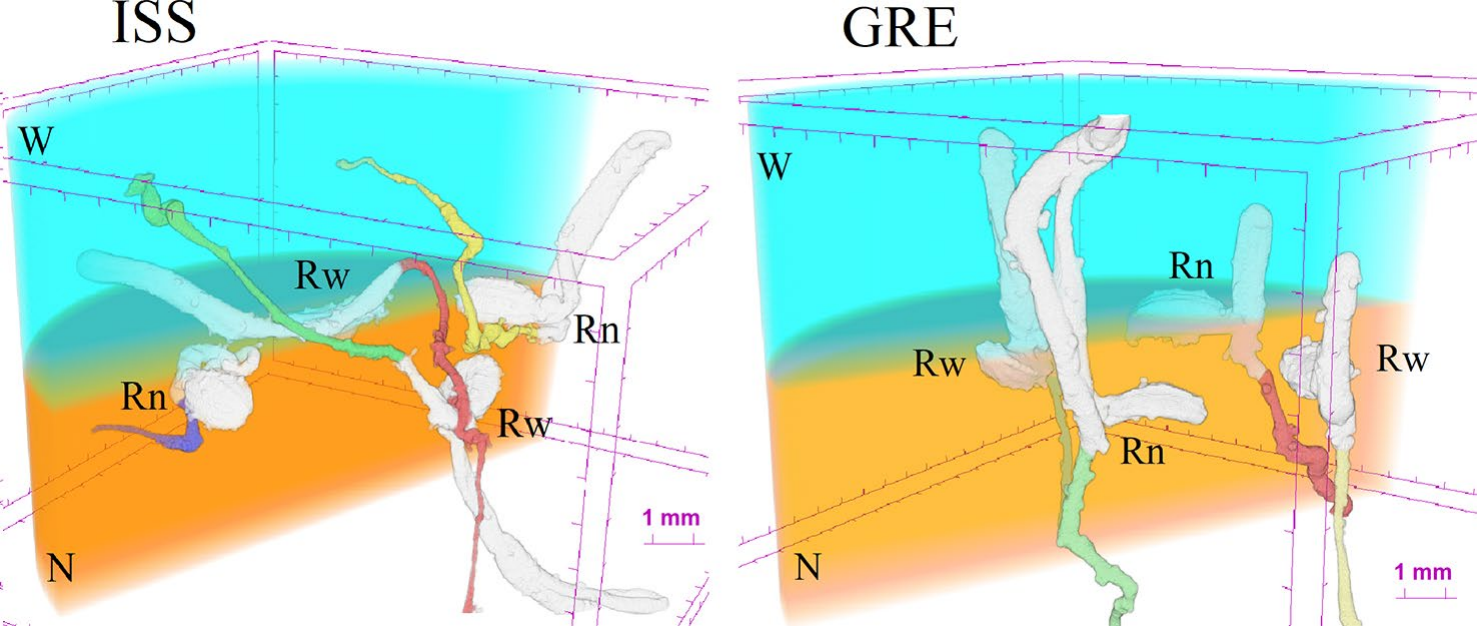
Examples of 3D reconstruction of the substrate assemblage with the four seeds germinated in the GRE and in the ISS. Rn and Rw refer to seed orientation facilitating the radicle growth toward the substrate with nutrients (N) or with water (W), respectively.

The X-ray microCT and the 3D image reconstruction not only allowed to define root growth orientation but also to address the morphometric comparison of carrot seedlings developed on the ISS and on Earth (**Table 2**). Considering that on the ISS 78% of the germinated seeds developed the root in the substrate with nutrient solution, for the sake of both statistical representativeness and homogeneity of the comparison, we chose to compare the morphology of the radicles in the nutrient solution for both cases, ISS and GRE. The first outcome of such a comparison (Supplementary Datasheet 1) showed that 83% of the seeds germinated in the GRE developed also hypocotyl, while in microgravity this occurred only to 44% of the seeds. Consequently, in order to compare morphometry of roots with similar degree of development, morphometric analyzes were restricted to the sprouts that showed the hypocotyl (see an example in **Figure 4**). The sprouts with hypocotyl showed no statistical differences in radicle volume, radicle diameter, radicle length and radicle tortuosity between roots developed in microgravity and in the GRE (**Table 2**; Supplementary Datasheet 2). In addition, considering sprouts from ISS with hypocotyl, a morphometric comparison was made between radicles grown in the substrate with nutrients and in that with water. No differences were found in morphometry of radicles except for the tortuosity (**Table 3**). The radicles grown in the substrate with water showed significantly higher tortuosity than those grown in the substrate with nutrients (Supplementary Datasheet 2). To be noted that the seed volume of the studied sprouts showed a very low variability with an average value of 3.0 mm^3^ and a standard error of 0.16 mm^3^ and no correlation was found between seed volume and radicle morphometric parameters (see Supplementary Datasheet 2.9).

**Table 2:** Morphometric parameters of radicles at the same development stage of *Daucus carota* grown in the substrate with nutrient in microgravity (ISS) and 1g (GRE) conditions.

	Root Volume mm^3^	Root Diameter mm	Root Length mm	Root Tortuosity
ISS	0.48 ± 0.07	0.32 ± 0.03	7.21 ± 1.71	1.22 ± 0.06
GRE	0.57 ± 0.08	0.36 ± 0.02	5.49 ± 0.35	1.16 ± 0.03

Each value represents the mean ± SE, n = 6 (ISS) and 10 (GRE).

**Table 3:** Comparison of morphometric parameters of roots of *Daucus carota* grown in the substrate with nutrient (N) or with water (W) only, in microgravity (ISS).

	Root Growth Direction	Root Volume mm^3^	Root Diameter mm	Root Length mm	Root Tortuosity
ISS	N	0.48 ± 0.07	0.32 ± 0.03	7.21 ± 1.71	1.22 ± 0.06 b
	W	0.55 ± 0.11	0.30 ± 0.02	9.64 ± 1.22	1.60 ± 0.17 a

Each value represents the mean ± SE, n = 6 (N) and 6 (W). Different letters in a column mean statistical difference between the average parameter (t-test one tail for root tortuosity).

## Discussion

The MULTITROP experiment was fully successful in reaching the goal of investigating the role of hydrotropism and chemotropism in root orientation in the absence of gravity stimulus. Results contribute to the international debate on the interaction between root tropisms. More specifically, despite the new evidence that hydrotropism does not function according to the Cholodny–Went theory, it is well established that the roots can exert a positive hydrotropism toward environments with greater water availability (Dietrich, 2018). On the other hand, root chemotropism is under debate and it can be often ambiguated with halotropism through which roots avoid high concentrations of salts (Galvan-Ampudia et al., 2013). Considerable chemical interactions occur in higher plants, especially for hormones and hormone-like substances released by microorganisms into the soil (Bilderback, 1985) and for chemotropic growth of the pollen tube (Hart, 1990). However, clear evidence of directional growth of roots toward a chemical compound are scarce. Data from our experiment performed on the ISS showed that disodium phosphate stimulates a positive chemotropism in primary roots of *D. carota*, and this tropic response overcome hydrotropism in microgravity. Such results support what observed by Newcombe and Rhodes at the beginning of the last century (Newcombe and Rhodes, 1904). From an ecological point of view, the search for anchoring and for water is a beneficial adaptive trait guiding the choices of radicle during seedling development (Bengough et al., 2011; Miyazawa et al., 2011). Although chemotropism of primary roots could be limited by the presence of endosperm in seeds that supports the demand for nutrients during germination, root development is influenced by nutrient supply and represents a useful adaptation in maximizing nutrient acquisition and tolerating unfavorable soils (Forde and Lorenzo, 2001; Ruiz Herrera et al., 2015). Interestingly, Newcombe and Rhodes (1904) showed that, beside the positive chemotropism toward disodium phosphate, roots of *L. albus* did not display a growth orientation toward various salts that are typically absorbed by plants as food. This raised the question whether a positive chemotropism of roots involves an adaptation for searching specific compounds, not necessarily absorbed as nutrients, and what could be the ecological explanation of this tropic response. Still, the attractive component of disodium phosphate stimulating the positive root chemotropism is unknown, and it could be either sodium or phosphoric acid ion. In this view, further research should reveal which chemical compounds can stimulate root chemotropism and what are the mechanisms underlying this phenomenon.

On Earth, gravitropism is dominant and typically masks tropisms guided by other stimuli (Takahashi, 1997; Takahashi, 2003). Accordingly, gravitropism overcome hydrotropism and chemotropism in primary roots of *D. carota* growing in 1g conditions which showed a downward orientation regardless of the presence/absence of nutrients in the substrate. Despite root growth follows the gravity vector on Earth, carrot seeds showed a preferential side for radicle protrusion. The choice of *D. carota* as species for the MULTITROP experiment was led by the need to satisfy technical constraints of the hardware and flight timeline (Aronne et al., 2018). The effect of morphological asymmetry of carrot seeds on the orientation of radicle protrusion interacted with root tropisms. However, this phenomenon proved with no doubt that chemotropism occurred in those seeds that reoriented growth toward disodium phosphate after the roots have protruded toward the substrate imbibed with water. As a general consideration, our results highlighted that attention should be paid to seed orientation when performing experiments on root tropisms. Nevertheless, an appropriate orientation of the seeds could be helpful also to optimize the design of cultivation systems for the space environment.

Results from MULTITROP experiment showed that germination percentage of carrot seeds in space was comparable with that on Earth. Likely, microgravity does not affect the germination rate of carrot seeds, but further tests should be carried out to confirm this result. Overall, *D. carota* can be considered a good candidate species for further experiments on plant biology and plant cultivation in space. However, within the same timeframe carrot seedlings in space developed in average less hypocotyls than on Earth. Differences in growth rate, slower in microgravity than in 1g condition, were reported also for soybean seedlings (De Micco and Aronne, 2008 De Micco et al., 2008).

The spaceflight poses many technical challenges to achieve scientific goals in a multiple-stress environment for plants (Aronne et al., 2018). Nevertheless, current and future research in space can make use of increasingly advanced tools and technologies. To the best of our knowledge, use of X-ray microCT imaging and 3D image analysis to obtain quantitative description of radicle morphology has never been implemented for germination experiments conducted on the ISS. In particular, the observation and analysis of the three-dimensional topology of the carrot radicles has allowed us to perform appropriate comparisons to better understand the differences between germination behavior on ISS and Earth, or in water vs nutrient solution, considering sprouts at similar development stages. From a morphological point of view, results from X-ray microCT showed that only root tortuosity was higher in roots developed in W compared to N in microgravity condition. Although no differences were highlighted in root morphology between roots developed in microgravity and in 1g condition, further investigations might verify the occurrence of ultrastructural aberrations in the space developed carrot seedlings as previously reported for soybean (De Micco et al., 2008).

Tropisms are crucial adaptive response to integrate external stimuli into plant architecture and can have significant agronomical applications. More and more insights come from experiments in space and the microgravity environment is revealing the magnitude of other tropisms, such as the greater phototropic response to blue light found in *A. thaliana* grown on the ISS (Millar et al., 2010). Similarly, in our experiment the chemotropic stimulus of disodium phosphate resulted more effective in orienting root growth in microgravity. In this framework, if the use of directional light could be an effective strategy to optimize plant development in space, it is not always applicable. Several plant species require dark condition to enhance seed germination, as the case of *D. carota* (Ozturk et al., 2009). In our experiment, seed germination occurred in dark conditions and this has also avoided confounding effects on the direction of root growth due to possible phototropism. In such a situation, specific nutritional solutions (e.g. sodium phosphate) can be used in plant cultivation to adjust root growth direction during germination. Overall, the efforts made to carry out the MULTITROP experiment on the ISS, despite the experimental constraints, also give new insights potentially useful to improve both plant research and cultivation in space.

Space exploration is a challenge of the current century and producing food is mandatory for long-term missions and future planet colonization (Wheeler, 2010). Bioregenerative life support systems, such as the Micro-Ecological Life-Support System Alternative (MELiSSA), are based on living organisms, including higher plants, to transform organic wastes and provide food, oxygen and water to the crew (Lasseur et al., 2010). Bioregenerative life support systems rely on biological processes and therefore require precise control to be efficient. Still, research is needed to predict plant growth in space, in particular in the light of upcoming missions to the Moon and Mars (Poulet et al., 2016). In this framework, space programs offer increasing research opportunities, while technological development of controlled-environment agriculture provides the possibility of more accurate experiments and cultivation in extreme environments (Gómez and Izzo, 2018).

## Conclusions

In our study it has been verified that the primary root of *D. carota* shows a positive chemotropism toward disodium phosphate in the absence of gravity stimulus. Besides increasing the understanding of plant tropisms, scientific outcomes of the MULTITROP experiment contribute to deepen our understanding of the role of different tropisms in plants and how they interact in shaping plant development aiming at the optimization of plant-based life support systems in space. Indeed, specific nutrient solutions or chemicals could be considered for plant experiments in microgravity where root growth needs to be directed into the root compartment. Still, further studies should be designed to investigate root chemotropism and its interaction with other tropisms by taking advantage of altered gravity conditions and using other chemical compounds.

## Data Availability Statement

The datasets generated for this study are available on request to the corresponding author.

## Author Contributions

GA conceived the idea of the MULTITROP experiment. LGI, LER, and GA performed the experiments and interpreted the results. GM did X-ray microCT scans and image processing. LGI did 3D image analysis. LG and GM provided the Supplementary material and interpreted and discussed results in **Tables 2** and **3**. LGI wrote the manuscript. GA, LER, SP, GM, and LG contributed to critical revision of the text.

## Funding

MULTITROP research has been financially supported by the agreement between ASI and the University of Naples Federico II, n. 2017-016-H.0. ASI coordinated the program and provided the access to the ISS and to the onboard resources thanks to the Memorandum of Understanding between ASI and NASA for the design, development, operation, and utilization of three mini pressurized logistic modules for the ISS.

## Conflict of Interest

The authors declare that the research was conducted in the absence of any commercial or financial relationships that could be construed as a potential conflict of interest.
