# Supplementary material for: Chemotropic vs Hydrotropic Stimuli for Root Growth Orientation in Microgravity
Source: Front Plant Sci. 2019 Nov 22;10:1547. doi: 10.3389/fpls.2019.01547 (PMC6883720; doi:10.3389/fpls.2019.01547)
Supplement: Supplementary file 1 [file DataSheet_1.pdf]

Supplementary Datasheet 1. Results of the observations and of the 3D morphometrical characterization of the radicles grown in the ISS and in the GRE.

	Seed N.	Seed orientation	Radicle growth direction	Radicle volume mm^3	Radicle mean diameter	Radicle length mm	Radicle tortuosity	Seedling development *	Seed size mm^3
ISS1A	1	Rw	N	0.673	0.232	15.520	1.27	H	2.67
ISS1A	2	Rw	W	0.706	0.316	11.560	1.42	H	4.09
ISS1A	3	Rn	W	0.925	0.316	13.337	1.75	H	3.34
ISS1A	4	Rn	N	0.250	0.318	4.177	1.30	H	2.29
ISS1B	1	Rn	N	0.195	0.322	1.853	1.10		
ISS1B	2	Rn	N	0.581	0.369	7.436	1.21		
ISS1B	3	Rn	N	0.063	0.318	0.909	1.08		
ISS1B		Rw							
ISS1C	1	Rw	W	0.687	0.364	7.327	1.46	H	2.37
ISS1C	2	Rn	N	0.475	0.301	6.921	1.13	H	3.47
ISS1C	3	Rn	N	0.144	0.290	2.506	1.18		
ISS1C	4	Rw							
ISS1D	1	Rn	N	0.688	0.438	4.770	1.44	H	2.50
ISS1D	2	Rw	W	0.467	0.270	11.158	1.52	H	2.94
ISS1D	3	Rw	W	0.306	0.236	9.244	2.34	H	3.31
ISS1D	4	Rn							
ISS2A	1	Rn	N	0.590	0.351	6.460	1.11		
ISS2A	2	Rw	N	0.150	0.279	2.717	1.14		
ISS2A	3	Rn	N	0.064	0.247	1.172	1.13		
ISS2A	4	Rw	N	0.036	0.273	0.61	1.03		
ISS2B	1	Rw	N	0.312	0.291	5.565	1.07	H	2.67
ISS2B	2	Rn	N	0.232	0.266	4.207	1.07		
ISS2B	3	Rn	N	0.072	0.267	1.575	1.05		
ISS2B	4	Rw							
ISS2C	1	Rw	N	0.509	0.333	6.292	1.14	H	2.31
ISS2C	2	Rw	N	0.212	0.387	1.428	1.36		
ISS2C	3	Rn	N	0.133	0.250	2.325	1.15		
ISS2C	4	Rn	N	0.029	0.248	0.737	1.15		
ISS2D	1	Rw	W	0.239	0.269	5.241	1.09	H	3.04
ISS2D	2	Rn	N	0.170	0.233	4.339	1.11		
ISS2D	3	Rn	N	0.077	0.265	1.018	1.09		
ISS2D	4	Rw							

	Seed N.	Seed orientation	Radicle growth direction	Radicle volume mm^3	Radicle mean diameter mm	Radicle length mm	Radicle tortuosity	Seedling development*	Seed size mm^3
GREA1A	1	Rw	N	0.646	0.350	7.340	1.15	H	2.63
GREA1A	2	Rn	N	0.381	0.364	4.137	1.11	H	3.50
GREA1A	3	Rn	N	0.348	0.299	5.693	1.08	H	1.97
GREA1A	4	Rw							
GREA1B	1	Rn	N	0.025	0.185	0.706	1.09		
GREA1B	2	Rn							
GREA1B	3	Rw							
GREA1B	4	Rw							
GREA1C	1	Rw	N	0.789	0.438	5.165	1.12	H	3.34
GREA1C	2	Rn	N	0.684	0.385	5.398	1.23	H	4.57
GREA1C	3	Rw	N	0.456	0.375	3.967	1.12	H	2.35
GREA1C	4	Rn	N	0.320	0.365	3.951	1.08		
GREA1D	1	Rn	N	1.068	0.480	6.224	1.22	H	4.38
GREA1D	2	Rn	N	0.712	0.372	6.887	1.41	H	1.63
GREA1D	3	Rw	N	0.322	0.312	4.789	1.06	H	3.61
GREA1D	4	Rw	N	0.263	0.258	5.299	1.10	H	2.93

* H = presence of hypocotyl in the sprouts
