# Supplementary material for: Chemotropic vs Hydrotropic Stimuli for Root Growth Orientation in Microgravity
Source: Front Plant Sci. 2019 Nov 22;10:1547. doi: 10.3389/fpls.2019.01547 (PMC6883720; doi:10.3389/fpls.2019.01547)
Supplement: Supplementary file 2 [file DataSheet_2.pdf]

Supplementary Datasheet 2. Results of the statistical analysis.

Datasheet 2.1 Results of the independent samples *t*-test and of Levene's test on the radicle volume (mm^3) data of the seedling with hypocotyl grown in the substrate with nutrient in GRE and in ISS.

T Test: Two Independent Samples

SUMMARY		Hyp Mean Diff		0
Groups	*Count*	*Mean*	*Variance*	*Cohen d*
GRE	10.0000	0.5670	0.0654	
ISS	6.0000	0.4845	0.0324	
Pooled			0.0536	0.3564

T TEST: Equal Variances				Alpha	0.0500				
	std err	*t-stat*	*df*	*p-value*	*t-crit*	*lower*	*upper*	*sig*	*effect r*
One Tail	0.1195	0.6901	14.0000	0.2507	1.7613			no	0.1814
Two Tail	0.1195	0.6901	14.0000	0.5014	2.1448	-0.1739	0.3389	no	0.1814

T TEST: Unequal Variances				Alpha	0.0500				
	std err	*t-stat*	*df*	*p-value*	*t-crit*	*lower*	*upper*	*sig*	*effect r*
One Tail	0.1093	0.7551	13.4685	0.2318	1.7709			no	0.2015
Two Tail	0.1093	0.7551	13.4685	0.4636	2.1604	-0.1535	0.3185	no	0.2015

Levene's test

H_0 = variances of all treatments are equal

F<*F*critical ↔ p-value>*α*

type	p-value
means	0.2279
medians	0.2312
trimmed	0.2279

Datasheet 2.2 Results of the independent samples *t*-test and of Levene's test on the radicle mean diameter (mm) data of the seedling with hypocotyl grown in the substrate with nutrient in GRE and in ISS.

T Test: Two Independent Samples

SUMMARY		Hyp Mean Diff		0
Groups	*Count*	*Mean*	*Variance*	*Cohen d*
GRE	10.0000	0.3633	0.0042	
ISS	6.0000	0.3188	0.0046	
Pooled			0.0044	0.6741

T TEST: Equal Variances				Alpha	0.0500				
	std err	*t-stat*	*df*	*p-value*	*t-crit*	*lower*	*upper*	*sig*	*effect r*
One Tail	0.0341	1.3053	14.0000	0.1064	1.7613			no	0.3294
Two Tail	0.0341	1.3053	14.0000	0.2128	2.1448	-0.0286	0.1176	no	0.3294

T TEST: Unequal Variances				Alpha	0.0500				
	std err	*t-stat*	*df*	*p-value*	*t-crit*	*lower*	*upper*	*sig*	*effect r*
One Tail	0.0345	1.2902	10.2836	0.1130	1.8125			no	0.3732
Two Tail	0.0345	1.2902	10.2836	0.2260	2.2281	-0.0323	0.1213	no	0.3732

Levene's test

H_0 = variances of all treatments are equal

F<*F*critical ↔ p-value>*α*

type	p-value
means	0.9157
medians	0.9090
trimmed	0.9157

Datasheet 2.3 Results of the independent samples *t*-test and of Levene's test on the radicle length (mm) data of the seedling with hypocotyl grown in the substrate with nutrient in GRE and in ISS.

T Test: Two Independent Samples

SUMMARY		Hyp Mean Diff		0
Groups	*Count*	*Mean*	*Variance*	*Cohen d*
GRE	10.0000	5.4899	1.1943	
ISS	6.0000	7.2075	17.5673	
Pooled			7.0418	0.6473

Levene's test

H_0 = variances of all treatments are equal

F<Fcritical ↔ p-value>α

type	p-value
means	0.0567
medians	0.1949
trimmed	0.0567

T TEST: Equal Variances				Alpha	0.0500				
	std err	*t-stat*	*df*	*p-value*	*t-crit*	*lower*	*upper*	*sig*	*effect r*
One Tail	1.3703	1.2534	14.0000	0.1153	1.7613			no	0.3176
Two Tail	1.3703	1.2534	14.0000	0.2306	2.1448	-4.6567	1.2215	no	0.3176

T TEST: Unequal Variances				Alpha	0.0500				
	std err	*t-stat*	*df*	*p-value*	*t-crit*	*lower*	*upper*	*sig*	*effect r*
One Tail	1.7457	0.9839	5.4112	0.1852	2.0150			no	0.3896
Two Tail	1.7457	0.9839	5.4112	0.3703	2.5706	-6.2049	2.7697	no	0.3896

Datasheet 2.4 Results of the independent samples *t*-test and of Levene's test on the radicle tortuosity data of the seedling with hypocotyl grown in the substrate with nutrient in GRE and in ISS.

T Test: Two Independent Samples

SUMMARY		Hyp Mean Diff		0
Groups	*Count*	*Mean*	*Variance*	*Cohen d*
GRE	10.0000	1.1615	0.0107	
ISS	6.0000	1.2223	0.0187	
Pooled			0.0136	0.5222

Levene's test

H_0 = variances of all treatments are equal

F<Fcritical ↔ p-value>α

type	p-value
means	0.2879
medians	0.2794
trimmed	0.2879

T TEST: Equal Variances				Alpha	0.0500				
	std err	*t-stat*	*df*	*p-value*	*t-crit*	*lower*	*upper*	*sig*	*effect r*
One Tail	0.0601	1.0113	14.0000	0.1645	1.7613			no	0.2609
Two Tail	0.0601	1.0113	14.0000	0.3290	2.1448	-0.1897	0.0681	no	0.2609

T TEST: Unequal Variances				Alpha	0.0500				
	std err	*t-stat*	*df*	*p-value*	*t-crit*	*lower*	*upper*	*sig*	*effect r*
One Tail	0.0647	0.9393	8.4538	0.1875	1.8595			no	0.3074
Two Tail	0.0647	0.9393	8.4538	0.3751	2.3060	-0.2101	0.0885	no	0.3074

Datasheet 2.5 Results of the independent samples *t*-test and of Levene's test on the radicle volume (mm^3) data of the seedling with hypocotyl grown in ISS in the substrate with nutrient (N) and in that with water only (W).

T Test: Two Independent Samples

SUMMARY		Hyp Mean Diff		0
Groups	*Count*	*Mean*	*Variance*	*Cohen d*
W	6.0000	0.5549	0.0693	
N	6.0000	0.4845	0.0324	
Pooled			0.0508	0.3122

Levene's test

H_0 = variances of all treatments are equal

F<*F*critical ↔ p-value>*α*

type	p-value
means	0.2197
medians	0.2261
trimmed	0.2197

T TEST: Equal Variances				Alpha	0.0500				
	std err	*t-stat*	*df*	*p-value*	*t-crit*	*lower*	*upper*	*sig*	*effect r*
One Tail	0.1302	0.5407	10.0000	0.3003	1.8125			no	0.1685
Two Tail	0.1302	0.5407	10.0000	0.6006	2.2281	-0.2197	0.3605	no	0.1685

T TEST: Unequal Variances				Alpha	0.0500				
	std err	*t-stat*	*df*	*p-value*	*t-crit*	*lower*	*upper*	*sig*	*effect r*
One Tail	0.1302	0.5407	8.8361	0.3009	1.8331			no	0.1790
Two Tail	0.1302	0.5407	8.8361	0.6018	2.2622	-0.2241	0.3649	no	0.1790

Datasheet 2.6 Results of the independent samples t-test and of Levene's test on the radicle mean diameter (mm) data of the seedling with hypocotyl grown in ISS in the substrate with nutrient (N) and in that with water only (W).

T Test: Two Independent Samples

SUMMARY		Hyp Mean Diff		0
Groups	*Count*	*Mean*	*Variance*	*Cohen d*
W	6.0000	0.2953	0.0021	
N	6.0000	0.3188	0.0046	
Pooled			0.0033	0.4063

Levene's test

H_0 = variances of all treatments are equal

F<*F*critical ↔ p-value>*α*

type	p-value
means	0.7206
medians	0.7403
trimmed	0.7206

T TEST: Equal Variances				Alpha	0.0500				
	std err	*t-stat*	*df*	*p-value*	*t-crit*	*lower*	*upper*	*sig*	*effect r*
One Tail	0.0333	0.7037	10.0000	0.2488	1.8125			no	0.2172
Two Tail	0.0333	0.7037	10.0000	0.4977	2.2281	-0.0977	0.0508	no	0.2172

T TEST: Unequal Variances				Alpha	0.0500				
	std err	*t-stat*	*df*	*p-value*	*t-crit*	*lower*	*upper*	*sig*	*effect r*
One Tail	0.0333	0.7037	8.7220	0.2497	1.8331			no	0.2318
Two Tail	0.0333	0.7037	8.7220	0.4994	2.2622	-0.0988	0.0519	no	0.2318

Datasheet 2.7 Results of the independent samples t-test and of Levene's test on the radicle length (mm) data of the seedling with hypocotyl grown in ISS in the substrate with nutrient (N) and in that with water only (W).

T Test: Two Independent Samples

SUMMARY		Hyp Mean Diff		0
Groups	*Count*	*Mean*	*Variance*	*Cohen d*
W	6.0000	9.6446	8.9035	
N	6.0000	7.2075	17.5673	
Pooled			13.2354	0.6699

Levene's test

H_0 = variances of all treatments are equal

F<Fcritical ↔ p-value>α

type	p-value
means	0.7702
medians	0.9982
trimmed	0.7702

T TEST: Equal Variances — Alpha 0.0500

	std err	*t-stat*	*df*	*p-value*	*t-crit*	*lower*	*upper*	*sig*	*effect r*
One Tail	2.1004	1.1603	10.0000	0.1364	1.8125			no	0.3445
Two Tail	2.1004	1.1603	10.0000	0.2729	2.2281	-2.2429	7.1172	no	0.3445

T TEST: Unequal Variances — Alpha 0.0500

	std err	*t-stat*	*df*	*p-value*	*t-crit*	*lower*	*upper*	*sig*	*effect r*
One Tail	2.1004	1.1603	9.0324	0.1379	1.8331			no	0.3602
Two Tail	2.1004	1.1603	9.0324	0.2758	2.2622	-2.3143	7.1887	no	0.3602

Datasheet 2.8 Results of the independent samples t-test and of Levene's test on the radicle tortuosity data of the seedling with hypocotyl grown in ISS in the substrate with nutrient (N) and in that with water only (W).

T Test: Two Independent Samples

SUMMARY		Hyp Mean Diff		0
Groups	*Count*	*Mean*	*Variance*	*Cohen d*
W	6.0000	1.5966	0.1761	
N	6.0000	1.2223	0.0187	
Pooled			0.0974	1.1992

Levene's test

H_0 = variances of all treatments are equal

F<Fcritical ↔ p-value>α

type	p-value
means	0.1278
medians	0.2533
trimmed	0.1278

T TEST: Equal Variances — Alpha 0.0500

	std err	*t-stat*	*df*	*p-value*	*t-crit*	*lower*	*upper*	*sig*	*effect r*
One Tail	0.1802	2.0771	10.0000	0.0323	1.8125			yes	0.5490
Two Tail	0.1802	2.0771	10.0000	0.0645	2.2281	-0.0272	0.7758	no	0.5490

T TEST: Unequal Variances — Alpha 0.0500

	std err	*t-stat*	*df*	*p-value*	*t-crit*	*lower*	*upper*	*sig*	*effect r*
One Tail	0.1802	2.0771	6.0518	0.0415	1.9432			yes	0.6451
Two Tail	0.1802	2.0771	6.0518	0.0831	2.4469	-0.0666	0.8152	no	0.6451

Datasheet 2.9 Pearson correlation coefficients and p-values between the seed volume (mm^3) data and the morphometric parameters of radicles.

	Radicle volume mm^3	Radicle mean diameter mm	Radicle length mm	Radicle tortuosity
Pearson coefficient	0.304	0.186	0.101	0.054
p-value	0.169	0.406	0.655	0.811
